# Diabetes Mellitus and Heart Failure

**DOI:** 10.3390/jcm10163682

**Published:** 2021-08-19

**Authors:** Filippos Triposkiadis, Andrew Xanthopoulos, Alexandra Bargiota, Takeshi Kitai, Niki Katsiki, Dimitrios Farmakis, John Skoularigis, Randall C. Starling, Efstathios Iliodromitis

**Affiliations:** 1 Department of Cardiology, University General Hospital of Larissa, 411 10 Larissa, Greece; andrewvxanth@gmail.com (A.X.); iskoular@gmail.com (J.S.); 2 Department of Endocrinology and Metabolic Diseases, University General Hospital of Larissa, 411 10 Larissa, Greece; abargio@yahoo.gr; 3 National Cerebral and Cardiovascular Center, Osaka 564-8565, Japan; t-kitai@kcho.jp; 4 Second Propedeutic Department of Internal Medicine, Medical School, Aristotle University of Thessaloniki, Hippokration Hospital, 54124 Thessaloniki, Greece; nikikatsiki@hotmail.com; 5 University of Cyprus Medical School, P.O. Box 20537, Nicosia 1678, Cyprus; dimitrios_farmakis@yahoo.com; 6 Kaufman Center for Heart Failure Treatment and Recovery, Heart, Vascular and Thoracic Institute, Cleveland Clinic, Cleveland, OH 44195, USA; starlir@ccf.org; 7 Second Department of Cardiology, National and Kapodistrian University of Athens, Attikon University Hospital, 12462 Athens, Greece; iliodromitis@yahoo.gr

**Keywords:** diabetes, heart failure, diabetic cardiomyopathy

## Abstract

Diabetes mellitus (DM) is a major risk factor for new-onset heart failure (HF) and vice versa. The pathogenesis of new-onset HF in DM is complex and has been largely attributed to the toxic cardiovascular effects of hyperglycemia and relevant metabolic abnormalities (diabetic cardiomyopathy) as well as the frequently coexisting morbidities such as hypertension (HTN), coronary artery disease (CAD), and diabetic nephropathy. In patients with type 1 DM (T1DM), HF develops in the setting of a dysregulated immune response, whereas in most patients with type 2 DM (T2DM), against a background of overweight/obesity. HF prevention in DM is feasible with rigorous treatment of cardiovascular risk factors and selective antidiabetic agents. Conversely, development of new-onset T2DM in HF (cardiogenic DM) is common and has been attributed to an increase in the resistance to insulin, especially in the skeletal muscle, liver, and adipose tissue as well as in diminished insulin secretory response to hyperglycemia by pancreatic β-cells. Cardiogenic DM further deteriorates cardiac dysfunction and adversely affects outcome in HF. Novel lifesaving medications employed in HF management such as sacubitril/valsartan and sodium glucose cotransporter 2 inhibitors (SGLT-2i) have a favorable metabolic profile and lower the incidence of cardiogenic diabetes. Whether mitigation of cardiogenic DM should be a treatment target in HF deserves further investigation.

## 1. Introduction

Diabetes mellitus (DM) is a major risk factor for several cardiovascular (CV) outcomes, including heart failure (HF) [1,2]. Epidemiological and observational studies have demonstrated that DM increases the risk for new-onset HF independent of other traditional risk factors. Each 1% increase in glycated hemoglobin A1c is associated with a 30% increase for risk of HF in Type 1 DM (T1DM) [3] and an 8% increase for HF risk in T2DM, the most common type of DM occurring in approximately 90% of diabetes patients [4]. The prevalence of cardiac dysfunction in people with T1DM and T2DM has been demonstrated to be as high as 14.5% and 35.0%, respectively [5,6].

Conversely, HF is a major risk factor for the pathogenesis of new-onset T2DM (cardiogenic diabetes). In a study including more than 100,000 patients with HF, the annual incidence of new-onset T2DM was approximately 2% in the first years after HF diagnosis and rose to around 3% after 5 years of HF duration [7].

The present work summarizes the mechanisms and prevention of HF development in DM and vice versa. The metabolic abnormalities underlying both disease states will be emphasized both in the context of pathogenesis and as treatment targets. Gaps of knowledge will also be discussed.

### 1.1. New-Onset Heart Failure in Diabetes

#### 1.1.1. Epidemiology

The Framingham Heart Study, which enrolled 5209 men and women who were followed up for 18 years, reported a higher incidence of HF in women (5-fold) than men (2.4-fold) with DM after adjustment for other risk factors, including age, coronary artery disease (CAD), and hypertension (HTN) [8]. A recent meta-analysis assessed the risk of new-onset and recurrent HF according to the presence or absence of DM. For new-onset HF, the pooled risk ratio (RR) 95% confidence interval (CI) of 69 studies that examined HF as a whole (both HF with preserved ejection fraction (HFpEF) and HF with reduced ejection fraction (HFrEF)) was 2.14 (1.96–2.34). The risk magnitudes between HFpEF and HFrEF were not significantly different in studies that examined the risk in both syndromes (*p* = 0.86), whereas for recurrent HF, the pooled RR (95% CI) of 38 studies was 1.39 (1.33–1.45). These data indicate that, in patients with DM, the magnitude of risk for new-onset HF and recurrent HF is large and DM is associated with a high incidence of both HFpEF and HFrEF [9].

#### 1.1.2. Pathogenesis

**Figure 1:**
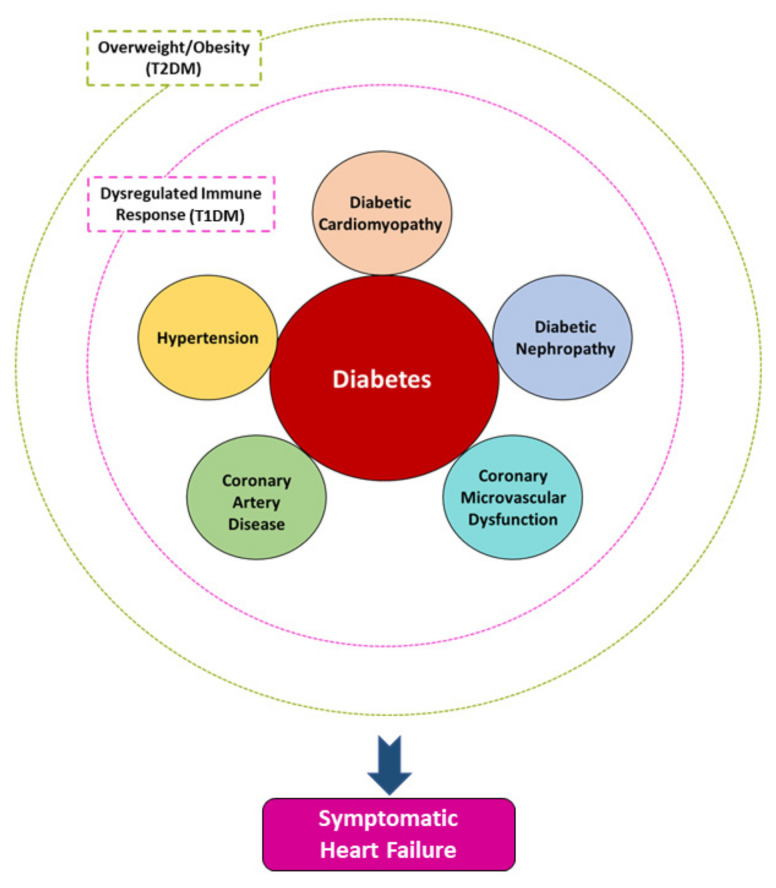
The pathogenesis of new-onset heart failure in diabetes mellitus (DM) is complex and has been largely attributed to the direct toxic effect of hyperglycemia and relevant metabolic effects on the myocardium (diabetic cardiomyopathy) as well as the frequently coexisting hypertension, coronary artery disease, coronary microvascular dysfunction, and diabetic nephropathy. In patients with type 1 DM, HF develops in the setting of a dysregulated immune response, whereas in most patients with T2DM, against a background of overweight/obesity.

The pathogenesis of new-onset HF in DM is complex and has been attributed to the direct toxic effect of hyperglycemia and relevant metabolic effects on the myocardium (diabetic cardiomyopathy) as well as the frequently coexisting HTN, CAD, coronary microvascular dysfunction (CMD), and diabetic nephropathy [10,11]. In patients with T1DM, HF develops in the setting of a dysregulated immune response, whereas in most patients with T2DM, against a background of overweight/obesity (Figure 1).

**Figure 2:**
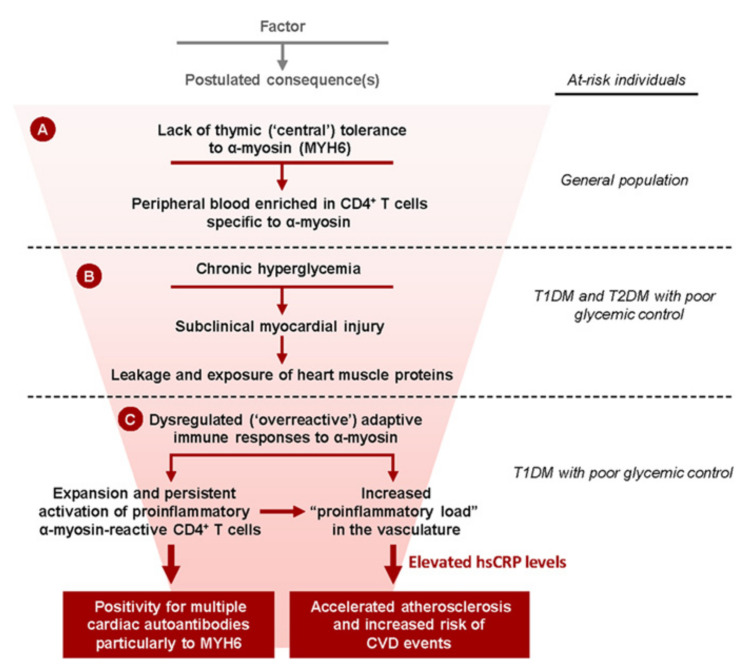
Proposed scheme of how chronic hyperglycemia is associated with cardiac autoimmunity and increased risk of cardiovascular disease (CVD) in patients with type 1 diabetes mellitus (T1DM). A: Absent thymic expression of the full-length α-cardiac myosin heavy chain (α-myosin; encoded by MYH6) is associated with high frequencies of CD4+ T cells specific to α-myosin in the peripheral blood of individuals in the general population. B: In both patients with T1DM and patients with type 2 diabetes mellitus (T2DM), chronic hyperglycemia causes subclinical myocardial injury, leading to leakage and exposure of heart muscle proteins, including α-myosin, to the immune system. C: In patients with T1DM, with poor glycemic control, the dysregulated adaptive immune system overreacts to myocardial injury, leading to the expansion of proinflammatory CD4+ T cells specific to α-myosin and the development of autoantibodies to MYH6 and other cardiac antigens. This proinflammatory state is associated with elevated levels of the inflammatory marker, high-sensitivity C-reactive protein (hsCRP), and increased risk for accelerated atherosclerosis and CVD events. Sousa, GR et al. Circulation 2019; 139:730–43 (Ref. [13]).

1. Immune dysregulation. T1DM results from the body’s immune system, which targets insulin-producing β-cells within the pancreatic islets. Patients with T1DM are also at increased risk of developing other autoimmune disorders, including cardiac autoimmunity [12]. Immune-mediated destruction of β-cells in pancreatic islets results in diminished or absent release of insulin and consequent hyperglycemia. Both in patients with T1DM and those with T2DM, chronic hyperglycemia causes subclinical myocardial injury, leading to leakage and exposure of heart muscle proteins, including α-myosin, to the immune system. However, in T1DM patients with poor glycemic control, the dysregulated adaptive immune system is overreactive to myocardial injury, leading to a build-up of proinflammatory CD4+ T cells specific to α-myosin and the development of autoantibodies to MYH6 and other cardiac antigens (Figure 2) [13]. This proinflammatory state leads to myocarditis resembling that seen in Chagas disease, and potentially a more generalized state of low-grade vascular inflammation, facilitating the development of atherosclerotic lesions and HF [14].
2. Obesity. Obesity is a major risk factor for the development of T2DM, HTN, CAD, and HF. Obesity alters the function of natriuretic peptides (NPs), which act as a defense mechanism against ventricular stress and the deleterious effects of cardiac volume and pressure overload. NPs act on the kidney promoting diuresis and natriuresis, inducing vasodilation, and protecting the heart from high preload and afterload, which can cause hypertrophy and fibrosis through activation of antifibrotic and antihypertrophic pathways [15]. In addition, NPs reduce the sympathetic tone and suppress renin and aldosterone secretion. Less recognized is the fact that NPs, by promoting adipocyte browning, lipolysis, lipid oxidation, and modulation of adipokine secretion, have emerged as key regulators of energy consumption and metabolism. NPR (NP receptor)-A signaling in skeletal muscles and adipocytes seems to be pivotal to the maintenance of long-term insulin sensitivity, which is disturbed in obesity and decreased glucose-tolerance states [16].

Obese individuals and T2DM patients suffer more frequently from HTN, left ventricular (LV) hypertrophy, and sympathetic activation than lean individuals, all of which may stimulate NP production [17]. However, it has been often shown that these subjects have lower circulating NPs than lean individuals. As a result of these crucial metabolic effects, NP deficiency is likely a pathophysiological link between obesity, arterial HTN, and insulin resistance (IR) or overt T2DM, all of which promote the development of HF [17,18].

Several types of LV remodeling have been described in obese individuals, including magnification (increased LV mass and volume, normal mass/volume ratio, and normal absolute wall thickness) [19,20], elevated mass-to-volume ratio due to a greater increase in LV mass than the LV end-diastolic volume [21], concentric LV remodeling, concentric LV hypertrophy, and eccentric LV hypertrophy [22,23,24]. It should be noted that although obesity has been linked with T2DM, emerging evidence suggests that obesity contributes to insulin resistance, dyslipidemia, and cardiometabolic complications in T1DM as well [25].

3. Diabetic cardiomyopathy. There are several definitions of diabetic cardiomyopathy. The 2013 European Society of Cardiology (ESC) Guidelines on diabetes, pre-diabetes, and CV diseases, developed in collaboration with the European Association for the Study of Diabetes (EASD), define diabetic cardiomyopathy as “a clinical condition diagnosed when ventricular dysfunction occurs in the setting of diabetes and in the absence of coronary atherosclerosis and hypertension” [26]. This definition was adopted by the 2018 position statement from the Heart Failure Association of the ESC [27].

Undoubtedly, myocardial dysfunction in the setting of DM without coexisting CV disorders may occur [28,29]. However, severe symptomatic myocardial dysfunction is usually associated with HTN and CAD, two major causes of HF [30,31,32,33]. Based on the above, throughout this manuscript the term diabetic cardiomyopathy will be used to designate the cardiac structural and functional abnormalities resulting from hyperglycemia and the relevant metabolic abnormalities (systemic insulin resistance and impaired cardiac insulin metabolic signaling, dyslipidemia) rendering the diabetic myocardium vulnerable to HF development [34,35].

### 1.2. Pathogenesis

Although the etiologies of T1DM and T2DM differ, systemic metabolic derangements such as hyperglycemia and dyslipidemia occur in both diseases [36]. Moreover, the increasing prevalence of overweight and obesity seems to be an emerging problem also in T1DM, and it has been suggested that obesity in the setting of T1DM may contribute to adverse CV outcomes and microvascular complications in adults with long-standing disease [37].

**Figure 3:**
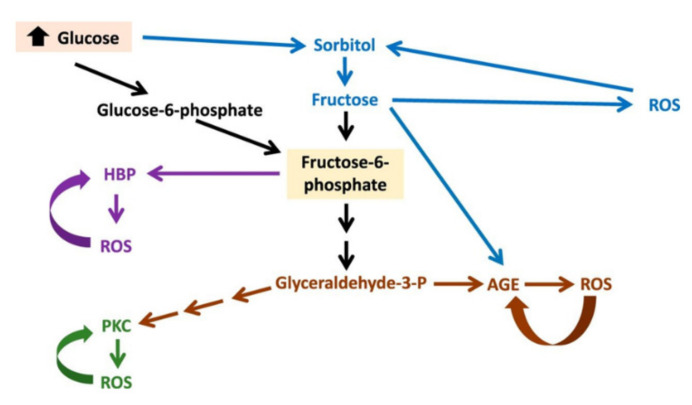
A model to demonstrate the metabolic vicious cycle whereby hyperglycemia-mediated stimulation of NOGPs fuels oxidative stress and further NOGP activation. The ROS produced by activation of the various NOGPs play a central role in fueling this vicious metabolic cycle. ROS produced by each pathway will further stimulate the respective NOGP as indicated, while also fueling the other NOGPs. The ROS produced will inhibit flux through the glycolytic pathway to further increase NOGP activation. In addition, higher ROS levels will deplete glutathione (GSH) and thus increase polyol pathway flux. Greater ROS availability will result in glucose autoxidation and lipid peroxidation that will generate increased AGEs. Increased ROS such as H2O2 can also directly cause activation of PKC. In addition, the polyol pathway plays a key role by activating other NOGPs. Here, it can generate fructose that can be further metabolized to produce AGEs and fructose-6-phosphate, which can lead to activation of the HBP, PKC, and AGEs. Mapanga, R.F., Essop MF. *Am J Physiol Heart Circ Physiol* 2016; 310: H153–H173 (Ref. [38]).

Hyperglycemia is associated with increased oxidative stress and glucose metabolic pathway perturbations, including hyperglycemia-induced activation of nonoxidative glucose pathways (NOGPs) (i.e., the polyol pathway, hexosamine biosynthetic pathway, advanced glycation end products (AGEs), and protein kinase C) [38]. There is a crucial interplay between NOGPs and a downstream confluence of adverse effects affecting cardiac endothelial cells, and thereby contributing to myocardial dysfunction. In this process, the AGE pathway appears to be a crucial mediator of hyperglycemia-induced adverse effects. In addition, a vicious metabolic cycle develops whereby hyperglycemia-induced NOGPs further incite their own activation by generating even more oxidative stress, thereby aggravating damaging effects on cardiac function (Figure 3).

**Figure 4:**
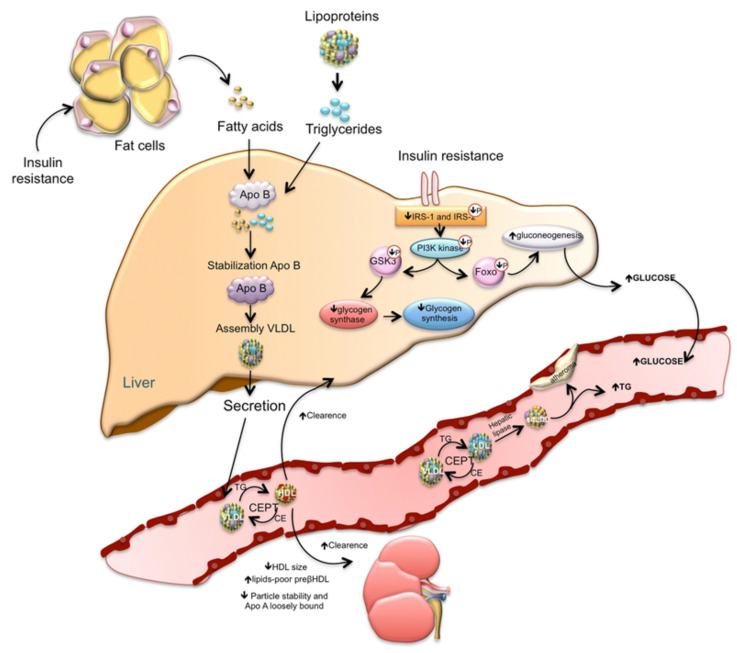
A simplified model of insulin resistance. The loss of suppressive effects of insulin on lipolysis in adipocytes increases free fatty acids. Increased free fatty acids flux to the liver stimulates the assembly and secretion of VLDL resulting in hypertriglyceridemia. Triglycerides (TG) in VLDL are transferred to both HDL and LDL through the action of cholesteryl ester transfer protein (CETP). This process results in a triglyceride-enriched HDL and LDL particle. Triglyceride-enriched HDL is more rapidly cleared from the circulation by the kidney, leaving fewer HDL particles to accept cholesterol from the vasculature. In the glucose metabolism, the insulin resistance results in decreased hepatic glycogen synthesis, owing to decreased activation of glycogen synthase, increased hepatic gluconeogenesis, and glucose delivery by the liver. Ormazabal, V. et al., Cardiovasc Diabetol 2018; 17:122 (Ref. [39]).

Insulin resistance (IR), which is a hallmark of T2DM, contributes to the development of CV disease (Figure 4). IR induces imbalance in glucose metabolism, contributing to the development of chronic hyperglycemia, which subsequently brings about oxidative stress and an inflammatory response leading to cellular damage [39]. IR can also affect systemic lipid metabolism, resulting in the development of dyslipidemia. Dyslipidemia, along with endothelial dysfunction, which can also be induced by aberrant insulin signaling, contribute to atherosclerotic plaque formation and myocardial damage generated by at least three different mechanisms: (1) alteration of signal transduction, (2) disturbed regulation of substrate metabolism, and (3) modified substrate delivery to the myocardium [39].

The next step in the pathophysiological process includes impairment of mitochondrial Ca2+ handling, autonomic neuropathy, activation of the renin–angiotensin–aldosterone system (RAAS), inflammation, stress of the endoplasmic reticulum, cardiomyocyte death, and microvascular dysfunction [40]. The myocardial response is characterized by a loss in metabolic flexibility, which is reflected in a greater reliance on glycolysis and ketone body oxidation as a source of energy and a decrease in the contribution of glucose oxidation to mitochondrial oxidative metabolism [41].

Hyperglycemia also induces myocardial fibrosis by stimulating several fibrogenic pathways including reactive oxygen species generation, augmentation of neurohormonal responses, activation of growth factor cascades, induction of pro-inflammatory cytokines and chemokines, synthesis of AGEs and mobilization of the AGE-major cell surface signal transduction receptor for AGE (receptor of AGE (RAGE)) axis, and upregulation of fibrogenic matricellular proteins [42]. These pathophysiological derangements promote cardiac stiffness, hypertrophy, and dysfunction [40].

### 1.3. Cardiac Structural and Functional Abnormalities

Early features of diabetic cardiomyopathy include LV hypertrophy and fibrosis, and cardiac dysfunction [43]. Traditionally, an early characteristic of diabetic cardiomyopathy has been considered diastolic dysfunction, identified as reduced diastolic filling pressures, prolonged isovolumetric relaxation, and increased atrial filling pressures in the absence of reduced LVEF. However, novel imaging modalities assessing myocardial deformation demonstrated the additional presence of subclinical systolic dysfunction with normal LVEF in the early stages of diabetic cardiomyopathy [44,45].

Left atrial (LA) structural, functional, and mechanical changes have an important role in the development of diabetic cardiomyopathy, and the extent of LA remodeling has a major prognostic role in patients with DM [46]. LA dysfunction is present early in diabetic cardiomyopathy. In a study which enrolled teenagers and young adults with normal LA volume, those with obesity and T2DM had lower E/A and higher E/e’, lower atrial reservoir, conduit, and booster strain, and worse reservoir and conduit strain rate compared to normal patients [47]. Likewise, right atrial (RA) and right ventricular (RV) abnormalities are also present and occur early. The Maastricht Study, a population-based cohort study, reported that individuals with prediabetes and DM compared to those with normal glucose metabolism (NGM) had lower RA volume index, lower RV diameter, smaller RV length, lower tissue doppler imaging (TDI) S’RV, lower TDI E’RV, and lower TDI A’RV [48]. These associations were largely not attributed to changes in LV structure, LV function, or pulmonary hypertension, suggesting that (pre)diabetes directly affects the RA and RV structure and function. Likewise, in the UK Biobank Cardiovascular Magnetic Resonance Substudy, a community cohort study which enrolled 3984 individuals without known CV disease and LVEF ≥ 50%, no difference was observed between individuals with DM and those without DM regarding, LVEF, LV mass, or RVEF, whereas LV and RV volumes (end-diastolic, stroke) as well as LA and RA emptying fractions were lower in DM [49].

**Figure 5:**
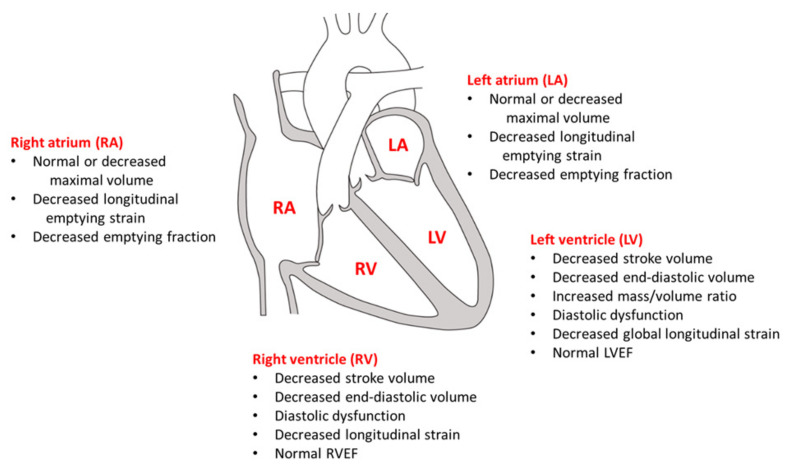
Early cardiac changes in morphology and function related to diabetes. Diabetes mellitus affects all 4 chambers of the heart. While right ventricular ejection fraction (RVEF) and left ventricular ejection fraction (LVEF) are preserved with no difference between diabetes mellitus and no diabetes mellitus, RV and LV chamber sizes are decreased. This occurs before increase in LV mass can be detected but is represented by an increased LV mass-to-volume ratio, suggesting early cardiac remodeling. Deformation imaging shows subtle impairment in LV function related to diabetes despite similar LVEF. The smaller ventricular volumes are accompanied by smaller right atrium (RA) and left atrium (LA) volumes. For both RA and LA, emptying function is impaired, which may represent an early marker of dysfunction occurring before impairments in LV or RV function. Figure based on data from: Steele, J.M. et al. *Cardiovasc Diabetol* 2020; 19:163 (Ref. [47]); Jensen, M.T. et al. *Circ Cardiovasc Imaging* 2019; 12:e009476 (Ref. [49]); Kumric, M., et al. *World J Diabetes* 2021; 12: 685–705 (Ref. [50]).

Thus, there is compelling evidence that DM results in early changes in all four cardiac chambers, which suggests that diabetic cardiomyopathy affects not only the LV but the heart globally (Figure 5).

4. Hypertension. HTN is present in many patients with DM. A recent analysis of US national surveys demonstrated that the prevalence of HTN in adults with DM was 76.3% or 66.0% according to the definitions of guidelines from the American College of Cardiology (ACC)/American Heart Association (AHA) and the American Diabetes Association (ADA), respectively [51]. An observational study, which included a cohort of teenagers and young adults who had been diagnosed with DM as a child or adolescent (*n* = 2018, follow-up period = 7.9 years), reported that patients with T2DM had a higher prevalence of HTN and increased arterial stiffness than those with T1DM [52]. Nevertheless, it is estimated that HTN affects around a third of patients with T1DM and that blood pressure control rates are disappointingly low [53]. Various common pathophysiological mechanisms contribute to the coexistence of HTN and DM, including, but not limited to IR, hyperinsulinemia, abnormal renal sodium handling, overactivation of RAAS, dysautonomia, inflammation, oxidative stress, and endothelial cell dysfunction [53,54].
5. Coronary artery disease. DM is a risk factor for CAD, independent of other major risk factors such as HTN, hyperlipidemia, and tobacco smoking. Baseline DM is associated with 2- to 3-fold increased rates of incident CAD, myocardial infarction (MI), and fatal CAD [55,56]. The Finnish study found that the seven-year incidence of MI in diabetic subjects with no history of prior MI was the same as that in nondiabetic subjects with a history of prior MI, giving rise to the concept of DM as a cardiovascular risk equivalent [57]. It is noteworthy that investigation of the relationship between T2DM and CAD has identified shared genes between T2DM and CAD with noticeable examples being the 9p21 locus, the IRS1 locus, and the LPL and ANGPTL4 genes, which are involved in triglyceride metabolism [58].

T1DM is associated with an almost threefold higher mortality than the general population, primarily driven by premature atherosclerosis both in men and women [59]. A recent meta-analysis estimated the standardized mortality ratio due to CV disease to be 5.7 for men and 11.3 for women with T1DM [60]. It is noteworthy that, by the age of 45 years, more than 70% of men and 50% of women with T1DM have developed coronary artery calcification [61].

Although the correlation between diabetes and CV disease is well established, the underlying mechanisms remain poorly understood. Complex interactions exist between the metabolic milieu present in DM and the formation of atherosclerotic lesions. In particular, the development of renal impairment markedly increases CV risk and there is an increasing recognition that unrecognized HF is accelerated by the presence of both renal and CV disease, further increasing morbidity and mortality [62].

6. Coronary microvascular dysfunction. The coronary microcirculation is a firmly regulated network with several associated physiological processes acting to match myocardial perfusion to metabolic demands [63]. Derangement of this mechanism, defined as CMD, is present in many DM patients and carries an increased risk of adverse CV clinical outcomes [64].

CMD can result in the inability of the coronary arteries to increase coronary blood flow (vasodilatory abnormality) and/or in a decrease in coronary blood flow (coronary microvascular spasm). Studies using CV magnetic resonance imaging, which can detect at the same time impaired myocardial microvascular perfusion and subclinical myocardial dysfunction in a single examination, have shown a correlation between impaired microvascular perfusion and subclinical myocardial dysfunction, suggesting a causal association between impaired coronary microcirculation and cardiac dysfunction [65,66].

In patients with DM, both structural and functional abnormalities, such as the endothelial dysfunction, may accompany the development of CMD without (Type 1 CMD) or with (Type 3 CMD) epicardial coronary artery disease [64]. The relationship between T2DM and CMD is bidirectional. Chronic hyperglycemia leads to significantly reduced endothelial-dependent and endothelial-independent coronary vasodilatation, whereas IR and hyperinsulinemia impair the microvascular endothelium-dependent coronary and skeletal vasodilatation by means of increased oxidative stress, inflammation, and dyslipidemia [67]. CMD, in turn, contributes to the progression of prediabetes to T2DM by reducing the delivery of insulin and glucose to skeletal muscles.

7. Diabetic nephropathy. Diabetic nephropathy is the most frequent cause of chronic kidney disease (CKD), representing a large and dire public health problem [68]. Diabetic nephropathy develops in the setting of a systemic, chronic proinflammatory state that contributes to vascular and myocardial remodeling, resulting in atherosclerotic lesions, vascular calcification, and vascular senescence as well as myocardial fibrosis and calcification of cardiac valves which mimic accelerated aging of the CV system [69,70]. Some studies suggest that the excess CV risk both in T1DM and T2DM is confined in patients with diabetic nephropathy [71,72]. Intensive glucose regulation decreases the risk of diabetic nephropathy and also suppresses the renin–angiotensin system (RAS) and is a significant treatment target both for the prevention and management of diabetic nephropathy [73]. However, hyperglycemia is often difficult to control in the CKD population, as several antihyperglycemic agents are contraindicated in CKD patients, and the pharmacokinetics of others, including insulin, change with declining glomerular filtration rate [74,75].

#### 1.3.1. Stages of Heart Failure Development in Diabetes

The progression of HF in DM is summarized in Table 1 [76,77,78]: (1) Stage A: the patient is asymptomatic and DM is a risk factor for the development of symptomatic HF; usually normal cardiac structure and function but occasionally subclinical cardiac structural and functional abnormalities due to diabetic cardiomyopathy; (2) Stage B: mild/moderate limitation of physical activity; structural and functional abnormalities are present due to diabetic cardiomyopathy but the LVEF is preserved; (3) Stage C: severe limitation of physical activity, severe structural cardiac abnormalities and depressed LVEF due to coexisting morbidities acting on top of diabetic cardiomyopathy; (4) Stage 4: symptoms at rest and death is imminent; there is biventricular refractory HF; this stage is also due to coexisting morbidities acting on top of diabetic cardiomyopathy.

**Figure 6:**
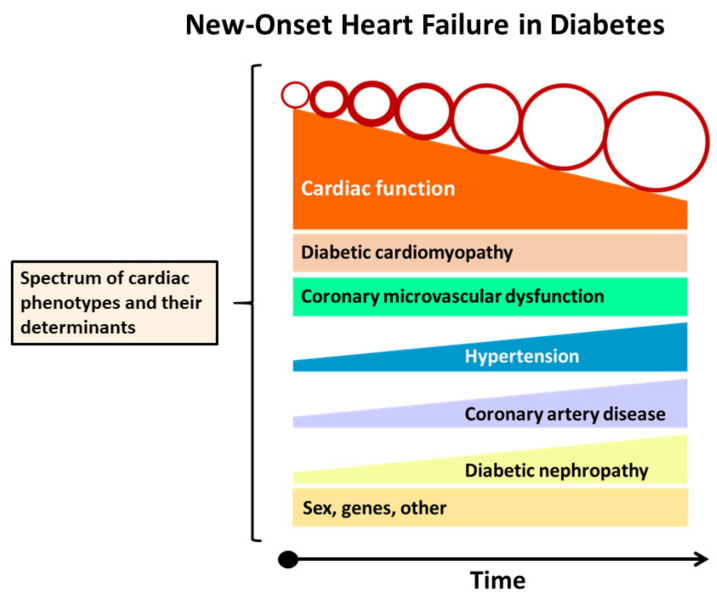
Spectrum of cardiac phenotypes and their determinants in new-onset heart failure (HF) in diabetes mellitus. New-onset HF in DM is a heterogeneous syndrome depending on diverse factors in which disease progression is associated with a dynamic evolution of functional and structural changes, leading to unique disease trajectories creating a spectrum of phenotypes with overlapping and distinct characteristics.

HF in DM was described as a dilated phenotype with eccentric LV remodeling and systolic LV dysfunction and a restrictive phenotype with concentric LV remodeling and diastolic LV dysfunction [79]. According to this view, the two phenotypes are not successive stages of HF in DM but evolve independently to, respectively, HFrEF and HFpEF. However, LVEF measurements are often inaccurate and variable, and a single LVEF value represents just a “snapshot” of ventricular performance, as it may improve or worsen during follow-up, it is often rate- and volume-dependent and exhibits significant interobserver and intraobserver variability [80,81]. Indeed, longitudinal changes in LVEF and transitions among HF phenotypes are common regardless of the presence or absence of DM, indicating that, in the modern era of HF management, the exclusive use of LVEF to categorize the pathophysiology of HF including HF in patients with DM might be non-comprehensive [82,83,84]. Instead, we propose that new-onset HF in DM is a heterogeneous syndrome depending on diverse factors in which disease progression is characterized by a dynamic evolution of functional and morphological derangements leading to unique disease trajectories, creating a spectrum of phenotypes with overlapping and distinct features (Figure 6) [84].

#### 1.3.2. Prevention of New-Onset Heart Failure in Diabetes

Current guidelines support a multifactorial approach in patients with DM, including lifestyle modification and medical treatment of hypertension and dyslipidemia [85]. Moreover, epidemiological studies suggest that higher levels of physical activity, cardiorespiratory fitness, and lower body mass index (BMI) are associated with lower risk of HF [86,87]. Bariatric surgery significantly lowers HF risk in patients with DM and morbid obesity [88,89].

Regarding glycemic control, although observational studies suggest that intensive glycemic control might reduce the incidence of HF at-risk individuals, this conjecture was not confirmed in randomized controlled trials which demonstrated that intensive glycemic control did not decrease the risk of hospital admission for HF [90,91]. However, in the past decade, several large CV outcome trials have provided data on the efficacy of metformin, glucagon-like peptide-1 (GLP1) receptor agonists (GLP1-RA), and sodium glucose cotransporter 2 inhibitors (SGLT-2i) to reduce major adverse CV and renal events in patients with T2DM [92].

Metformin is an insulin-sensitizing agent that works mainly by reducing gluconeogenesis and opposing glucagon-mediated signaling in the liver and, to a lesser extent, by increasing glucose uptake in skeletal muscle [93]. Metformin was the first glucose-lowering agent reported to improve CV outcomes in the UK Prospective Diabetes Study (UKPDS) and therefore became the standard of care [94]. However, as this clinical trial was conducted more than 20 years ago, it is difficult to compare the efficacy of metformin with that of novel agents such as the GLP1-RA and the SGLT-2i. Nevertheless, several lines of evidence support an antiatherosclerotic effect and reduced risk of adverse CV outcomes in T2DM as long as appropriate dose adjustments are made, and use is confined in patients with eGFR >30 mL/min/1.73 m^2^ [95].

GLP1-RA are non-insulin injection antidiabetic drugs (liraglutide, lixisenatide, albiglutide, dulaglutide, semaglutide, and loxenatide) which mimic the intestinal hormone incretin and bind its receptor. GLP-1 is released from the distal ileum and colon within minutes after a meal and, while it does augment glucose-dependent insulin synthesis and secretion, it has also been shown to decrease glucagon secretion, increase glucose uptake and glycogen synthesis in peripheral tissues, delay gastric emptying, and increase satiety [96]. Liraglutide, subcutaneous semaglutide, albiglutide and dulaglutide have all shown significant decreases in adverse CV events, whereas lixisenatide, weekly exenatide, and oral semaglutide have shown non-inferiority but not superiority [96].

SGLT-2i (empagliflozin, canagliflozin, dapagliflozin, and ertugliflozin) are a new class of antidiabetic drugs which reduces hyperglycemia through inhibition of glucose reabsorption in the renal proximal tubules. SGLT are active glucose co-transporters in the renal tubules preventing glycosuria by means of filtered glucose reabsorption along with sodium into the circulation. SGLT2, which is located almost exclusively in the S1 segment of the proximal renal tubules, carries out 90% of glucose resorption, whereas SGLT1, which is located distally in the S3 segment of the proximal tubule, reabsorbs the remaining 10% of filtered glucose [97]. SGLT1 is also located in the small intestines, heart, skeletal muscles, pancreas, and salivary glands. In T2DM, the compensatory upregulation of SGLT1/2 increases glucose reabsorption and contributes to maladaptive processes of energy conservation and exacerbation of hyperglycemia. Selective inhibition of SGLT2 results in approximately 80 g/day of urinary glucose excretion.

**Figure 7:**
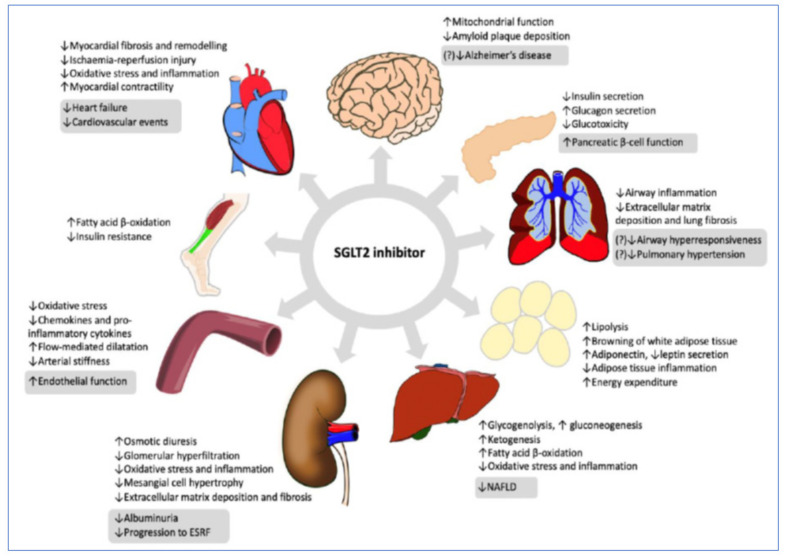
Sodium glucose cotransporter 2 (SGLT2) inhibition has pleiotropic effects on multiple organ systems. Hoong, C.W.S. et al. Endocrinology 2021 (Ref. [97]).

Despite SGLT2 localization predominantly in the renal tubules, SGLT-2i have important systemic effects [97]. In this regard, clinical studies have shown that SGLT-2i not only improve glycemic control but additionally reduce major adverse CV events and hospitalization for heart failure (HF) and improve outcome in CKD, both in patients with DM and those without DM [98,99]. Mechanisms that explain the protective effects of SGLT-2i include diuresis/natriuresis, blood pressure lowering, erythropoiesis, optimization of cardiac energy metabolism, inflammation reduction, inhibition of the sympathetic nervous system activity, prevention of cardiac remodeling, prevention of ischemia/reperfusion injury, inhibition of the Na+/H+-exchanger, reduction in hyperuricemia, increase in autophagy and lysosomal degradation, decrease in epicardial fat mass, increase erythropoietin levels, increase in circulating pro-vascular progenitor cells, decrease in oxidative stress, and improvement of vascular function (Figure 7) [100]. In patients with T2DM and recent hospitalization for decompensated HF, sotagliflozin (an SGLT2 and SGLT1 inhibitor), initiated before or shortly after discharge, resulted in a significantly lower total number of deaths from CV causes and hospitalizations and urgent visits for HF than placebo (SOLOIST-WHF trial) [101]. Whether SGLT-1 inhibition adds benefit to SGLT-2 inhibition in patients with T2DM is currently under investigation [102].

The American Diabetes Association (ADA)–European Association for the Study of Diabetes (EASD) recommend metformin for all patients with newly diagnosed T2DM [103], whereas the European Society of Cardiology (ESC) guidelines indicate that SGLT-2i or GLP1-RA should be offered first in the presence of high or remarkably high CV risk or of CV disease [104]. This difference may not be significant in clinical practice, since patients at high risk of cardiorenal disease should be treated with SGLT-2i or GLP1-RA independent of HbA1c, and most patients with T2DM rapidly require combination therapy [105]. However, when selecting an agent, one should bear in mind that, although GLP1-RA have been considered effective in the reduction of atherosclerotic events, including myocardial infarction and stroke, whereas SGLT-2i in the reduction of incident HF [106,107,108], the latter may be superior than the former in DM patients: 1) with more than three cardiovascular risk factors without established CV disease or CKD, 2) with established CV disease and CKD, 3) committed to further reducing their risk for CVD and CKD outcomes [109].

### 1.4. New-Onset Diabetes (Cardiogenic Diabetes) in Patients with Heart Failure

#### 1.4.1. Epidemiology

In the Bezafibrate Infarction Prevention study, the incidence of T2DM among HF patients increased in a stepwise manner from 13% in New York Heart Association (NYHA) Class I to 20% in NYHA Class III during a mean follow-up of 7.7 years, and being in NYHA Class III was an independent risk factor for the development of T2DM [110]. In the Danish nationwide register including 139,264 HF patients (29,078 with prevalent T2DM at baseline) [111], a total of 11,819 (8%) developed incident T2DM and the median time interval between HF diagnosis, and new-onset T2DM diagnosis was 4.1 years (IQR:1.5; 5.8). The development of incident T2DM in patients with HF increased the risk of HF hospitalization and mortality by threefold.

#### 1.4.2. Pathogenesis

The clinical expression of T2DM usually follows a decrease in the responsiveness (increase in the resistance) to insulin (especially in skeletal muscle, liver, and adipose tissue) and a decrease in insulin secretory response to hyperglycemia by pancreatic β cells.

Chronic HF is an IR state. IR occurs in more than half of HF patients and is associated with impaired cardiac innervation (decreased early and late heart-to-mediastinum ratios and washout rate in 123I-MIBG imaging), indicating overactivity of the cardiac sympathetic nervous system [112]. Moreover, IR is associated with RAAS overactivity [14] due to impaired insulin action [113]. Although the exact underlying cause of IR in HF has not been fully elucidated, several major mechanisms have been proposed, including oxidative stress, inflammation, insulin receptor mutations, endoplasmic reticulum stress, and mitochondrial dysfunction [114].

**Figure 8:**
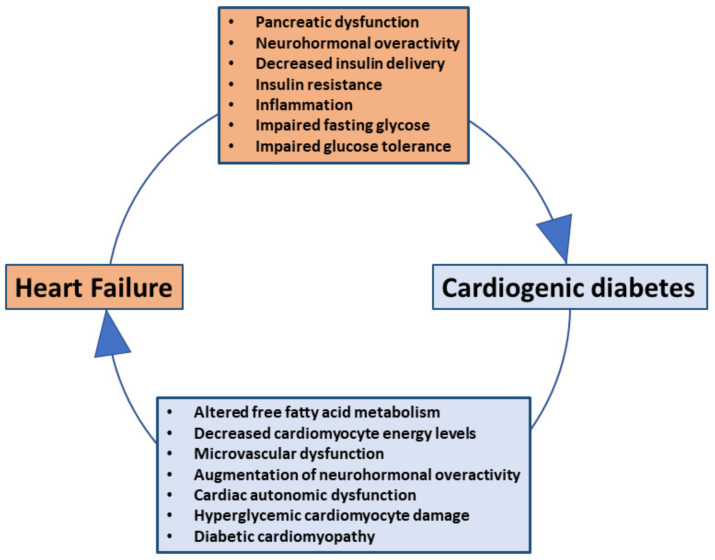
Heart failure (HF) leads to the development of cardiogenic diabetes, which subsequently adversely affects cardiac function and HF outcome.

IR results in reduced skeletal muscle glucose uptake, diminished inhibition of hepatic glucose output, and increased lipolysis and free fatty acid (FFA) release from adipose tissue. The elevated free fatty acid (FFA) levels increase β-cell mass and insulin secretion to compensate for insulin insensitivity. Over time, however, the metabolic consequences of IR (hyperglycemia and hyperlipidemia) in genetically susceptible individuals may result in “β-cell failure” characterized by reduced β-cell number, dysfunction, and compromised identity thus promoting T2DM [115]. The mechanisms by which HF causes cardiogenic DM and the latter further worsens cardiac function and adversely affects outcome in HF are summarized in Figure 8.

#### 1.4.3. Prevention of New-Onset Diabetes in Patients with Heart Failure

Traditional medications used in the treatment of HF have diverse metabolic effects. Diuretics are associated with IR and increased serum uric acid levels which may contribute to abnormal glucose tolerance [116]. B-blockers are a heterogenous class of agents. Vasodilating b-blockers (e.g., carvedilol) have a more favorable metabolic profile than the non-vasodilating beta-blockers (e.g., metoprolol) [117,118]. Angiotensin-converting enzyme inhibitors/angiotensin receptor blockers may prevent or delay the onset of DM [119], whereas eplerenone has no effect on new-onset DM in patients with HF [120]. Sacubitril/valsartan has a favorable antidiabetic profile. It improves peripheral insulin sensitivity compared with amlodipine in obese hypertensive patients who are at high risk to develop DM [121] and achieves a greater HbA1c reduction compared with enalapril in patients with HF [122].

**Figure 9:**
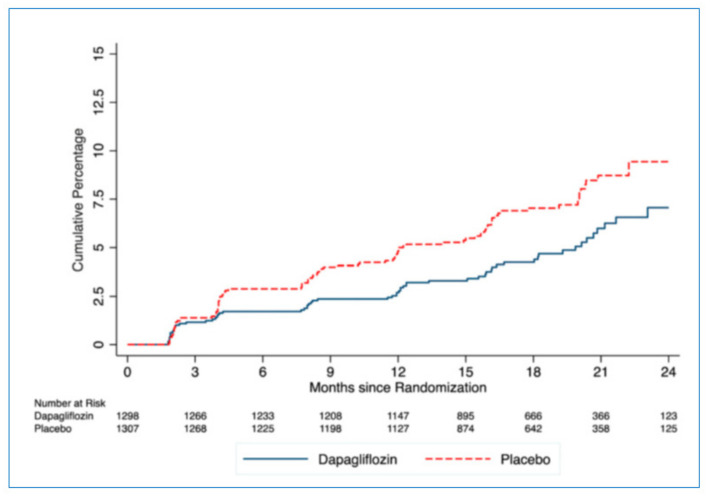
Incidence of new-onset type 2 diabetes mellitus (T2DM) in dapagliflozin vs. placebo groups in the Dapagliflozin and Prevention of Adverse Outcomes in Heart Failure (DAPA-HF) trial. The hazard ratio (HR) for incident T2DM in the dapagliflozin group compared with placebo was 0.68 (95% CI 0.50–0.94; P50.019), with an early divergence of the event curves. Inzucchi, S.E. et al., *Diabetes Care* 2021; 44:586–594 (Ref. [126]).

SGLT-2i are novel agents that significantly improve outcomes in patients with established HF, regardless of LVEF and the presence or absence of T2DM [123,124]. Further, SGLT-2i reduce the risk for new-onset T2DM in HF. In the Empagliflozin Outcome Trial in Patients with Chronic Heart Failure and a Reduced Ejection Fraction (EMPEROR-Reduced), the SGLT-2i empagliflozin was associated with a 14% decrease in the risk for the onset of new T2DM in patients with prediabetes compared with placebo [125]. Likewise, in the Dapagliflozin and Prevention of Adverse Outcomes in Heart Failure (DAPA-HF) trial, dapagliflozin led to a 32% reduction in T2DM incidence during a median follow-up of 18 months (Figure 9) [126]. These findings are in accordance with the fact that the administration of SGLT-2i decreases IR and improves β-cell function [127].

#### 1.4.4. Gaps in Knowledge and Future Perspectives

Different imaging modalities such as magnetic resonance imaging (MRI), speckle tracking echocardiography (STE) and nuclear imaging have been successfully employed in the recognition of early myocardial changes of diabetic cardiomyopathy. Nevertheless, many of these advanced techniques are not routinely used by clinicians and are limited to research. It is likely that these techniques will be used more often in the future as some of the features identified by them in diabetic cardiomyopathy (e.g., impaired myocardial perfusion, myocardial fibrosis) may become treatment goals requiring monitoring. There is also increasing focus on combining imaging techniques for better phenotypic classification.

Besides imaging, other important issues complicate the assessment of the early stages of diabetic cardiomyopathy, especially in T2DM. The presence of a cardiac dysfunction in a young normoweight patient with T1DM but without HTN, CAD or CKD suggests the presence of diabetic cardiomyopathy. However, the same level of cardiac dysfunction in an obese patient with T2DM matched for the other characteristics cannot be exclusively attributed to diabetic cardiomyopathy since obesity per se may contribute to the development of similar cardiac dysfunction (obesity cardiomyopathy) [128]. To complicate things further, severe cardiac dysfunction with reduced LVEF is rare in a diabetic patient in the absence of HTN, CAD or CKD and even in patients with DM and reduced LVEF, but without coexisting morbidities, one may not always be certain whether the reduced LVEF is due to DM or vice versa (cardiogenic diabetes) [129,130,131]. Finally, it may be difficult for a clinician to determine whether cardiac dysfunction with reduced LVEF is due to DM or is actually a dilated cardiomyopathy with another cause. Typical symptoms combined with an elevated B-type natriuretic peptide indicate the presence of HF but are not specific. Likewise, an abnormal echocardiogram or cardiac MRI can identify structural cardiac abnormalities, but not that these are due to DM.

Although plasma inflammatory biomarkers (e.g., interleukin-6 and C-reactive protein) and macroalbuminuria [132], as well as red blood cell distribution width (RDW) [133], have been shown to be predictors of HF their ability to predict HF in the individual patient with DM is limited. Although it has been recently suggested that lncRNA, sST2 and galectin-3 may be promising biomarkers for diabetic cardiomyopathy detection as their plasma/serum levels correlate with the early stages of diabetic cardiomyopathy, further validation studies are necessary [50]. Thus, there is an urgent need for diagnostic approaches that incorporate biomarkers unique to the diabetic cardiomyopathic process [134]. Successful implementation of this approach will additionally require the use of multimodality imaging, rendering feasible the determination of the extent of reversible myocardial interstitial fibrosis driven by inflammation or the acquisition of quantitative information on metabolic and molecular processes relevant to DM and diabetic cardiomyopathy [135,136].

Regarding prevention, there are several uncertainties concerning the use of antidiabetic agents to mitigate CV complications in DM patients. These include the potential additional benefit when combining SGLT-2i with GLP1-RA, the benefits and harms of using SGLT-2i inhibitors in CKD patients with severely impaired eGFR (i.e., <20 or 30 mL/min/1.73 m^2^), the lack of validated tools assessing the baseline risk for all CV outcomes according to ethnicity, race, and geography, and the lack of studies assessing the effect of different diabetes drugs on life quality [109]. Finally, whether inhibition of the development of new-onset DM in HF should be a treatment target deserves urgent investigation.

## 2. Conclusions

There is a strong bidirectional association between DM and HF. DM is an established risk factor of HF and HF can cause DM (cardiogenic diabetes). A number of pathophysiological mechanisms have been implicated in the pathogenesis of new-onset HF in DM including diabetic cardiomyopathy, HTN, MCD, CAD, and CKD, usually acting against a background of immune dysregulation in T1DM and obesity in T2DM. Cardiac dysfunction in patients with DM is represented by a spectrum of phenotypes ranging from mild structural/functional abnormalities causing no symptoms to severe symptomatic eccentric hypertrophy, usually accompanied by HTN, CAD, or CKD. The best antidiabetic medications to prevent HF development in T2DM patients are SGLT-2i. It is noteworthy that the same agents, which currently represent a powerful tool in the management of HF regardless of the LVEF or the presence or absence of DM, are powerful inhibitors of the development of new-onset T2DM in HF patients.

## Figures and Tables

**Table 1:** Stages of newonset heart failure in patients with diabetes.

Features	Stage			
	A	B	C	D
**Diabetic cardiomyopathy**	**Present**	**Present**	**Present**	**Present**
**Coexisting morbidities** **(hypertension, coronary artery disease (CAD), chronic kidney disease (CKD))**	**Absent**	**Absent**	**Present**	**Present**
**Mechanisms**	**Metabolic disturbances (hyperglycemia, insulin resistance, hyperinsulinemia) leading to cardiac steatosis, microvascular dysfunction, increased ROS, and inflammation **	**Stage A plus myocardial necrosis, interstitial fibrosis, capillary microaneurysms, coronary microvascular rarefaction, and loss of cardiac metabolic flexibility**	**Stage B plus cardiovascular impairment due to coexisting morbidities**	**Stage C plus increased severity of coexisting morbidities** **(e.g., diffuse CAD and/or severe CKD)**
**Left ventricular remodeling**	**Absent ** **Occasionally concentric remodeling or hypertrophy**	**Concentric remodeling or hypertrophy**	**Concentric hypertrophy or indeterminate hypertrophy (magnification) or eccentric hypertrophy**	**Eccentric ** **hypertrophy**
**Cardiac function**	**Normal or mild diastolic dysfunction and mild decrease in systolic strain of both atria and ventricles **	**Diastolic dysfunction, decreased systolic strain** **Right and left atrial and ventricular involvement**	**Severe diastolic dysfunction** **Decreased LVEF** **Pulmonary hypertension**	**Biventricular refractory** **heart failure**
**NYHA ** **functional class**	**Asymptomatic, no limitation of physical activity (NYHA I)**	**Symptoms occurring during ordinary physical activity** **Slight/moderate limitation of physical activity (NYHA II)**	**Symptoms occurring during minimal physical activity.** **Marked limitation of physical activity (NYHA III)**	**Symptoms occurring at ** **rest. Unable to carry out ** **any physical activity** **without discomfort** **(NYHA IV)** **Death**

HTN, hypertension; CAD, coronary artery disease; CKD, chronic kidney disease; ROS, reactive oxygen species; LVEF, left ventricular ejection fraction; NYHA, New York Heart Association.
